# Effectiveness and Nephrotoxicity of Intravenous Polymyxin B in Carbapenem-Resistant Gram-Negative Bacterial Infections Among Chinese Children

**DOI:** 10.3389/fphar.2022.902054

**Published:** 2022-05-27

**Authors:** Xuedong Jia, Zhao Yin, Wan Zhang, Conghui Guo, Shuzhang Du, Xiaojian Zhang

**Affiliations:** ^1^ Department of Pharmacy, The First Affiliated Hospital of Zhengzhou University, Zhengzhou, China; ^2^ The Precision Clinical Pharmacy Key Laboratory of Henan, Zhengzhou, China

**Keywords:** effectiveness, nephrotoxicity, polymyxin B, carbapenem-resistant gram-negative bacterial infections, Chinese children

## Abstract

**Background:** No clinical study on the use of polymyxin B in Chinese children has been reported, thus making it difficult for pediatric clinicians to rationally select these drugs.

**Methods:** A retrospective analysis of children treated with polymyxin B during hospitalization in a hospital from June 2019 to June 2021 was conducted to analyze its effectiveness and the incidence of acute kidney injury (AKI) during treatment with polymyxin B.

**Results:** A total of 55 children were included in this study, and the results showed that the intravenous polymyxin B-based regimen had an effective rate of 52.7% in the treatment of Carbapenem-resistant Gram-negative bacterial (CR-GNB) infection in children. The results of the subgroup analysis showed that the course of treatment was longer in the favorable clinical response group than in the unfavorable outcome group (*p* = 0.027) and that electrolyte disturbances in children during the course of treatment could lead to unfavorable clinical outcomes (*p* = 0.042). The risk of incidence of AKI during treatment was 27.3%, and the all-cause mortality rate in the children on their discharge from the hospital was 7.3%.

**Conclusion:** Polymyxin B can be used as a salvage therapy for CR-GNB infection in children when no other susceptible antibiotics are available, and the monitoring of kidney function should be strengthened.

## Introduction

Due to the irrational use or even abuse of antibiotics in recent years, bacterial resistance has increased constantly, and bacterial resistance to carbapenems, previously known as the last line in the battle against drug-resistant Gram-negative bacterial (GNP) infections, has also become a serious problem (Ding et al., 2019; Chiotos et al., 2020; Gao et al., 2020). The results from the China Antimicrobial Surveillance Network (CHINET) have shown that the resistance rate of Gram-negative bacteria to carbapenem antibiotics is increasing year on year, and there has been a growing threat of antimicrobial resistance, posing a great challenge to human anti-infection treatment (Hu et al., 2019; Han et al., 2020; Liao et al., 2020). Polymyxins, an old class of antibiotics, have been gradually replaced by safer antibiotics due to its higher incidence of nephrotoxicity and neurotoxicity (Nang et al., 2021; Soman et al., 2021). However, this kind of drug has been reintroduced into clinical practice in recent years for the treatment of drug-resistant Gram-negative infections, as they still maintain a high activity to carbapenem-resistant Gram-negative bacilli (CR-GNB) commonly isolated in clinical practice (Pogue et al., 2020; Satlin et al., 2020).

Currently, polymyxin B and polymyxin E (colistin) are commonly recommended for the treatment of extensively drug-resistant or pandrug-resistant Gram-negative bacilli in clinical (Tsuji et al., 2019; Yang et al., 2021). However, only two case series studies have analyzed the clinical efficacy and safety of polymyxin B in children (Saleem et al., 2011; Siddiqui et al., 2014), both studies had small sample sizes (Fourteen and seven patients, respectively) and differed in the infection type of patients, the dosing schedule of polymyxin B and the outcomes of the studies, so the results of both studies have shown large differences in the efficacy (57.1 vs. 100%) and incidence of acute kidney injury (AKI) (21.4 vs. 0%). Therefore, there is an urgent need for studies with large samples to further explore the effectiveness and safety of polymyxin B in children. In 2018, polymyxin B was relaunched in the Chinese market for use (Yu et al., 2020), The indications and methods of use of the drug in children are not clearly specified in the National Medical Products Administration (NMPA) approved drug insert. And thus far, no clinical research data on the use of polymyxin B in Chinese children have been reported. In this study, a retrospective analysis of clinical data on the use of intravenous polymyxin B in children in a hospital over 2 years was carried out to summarize its efficacy and nephrotoxicity and provide clinicians with some reference for the rational use of such drugs.

## Methods

### Study Design

This study was approved and supervised by the Ethics Committee of the First Affiliated Hospital of Zhengzhou University (Grant No. 2021-KY-0063-002). This study focused on children who were discharged from the First Affiliated Hospital of Zhengzhou University (bed capacity >8500), currently the largest hospital in China, between June 2019 and June 2021 and were treated with intravenous polymyxin B for carbapenem-resistant Gram-negative bacilli (CR-GNB) infection during their hospitalization period. All children (<18 years old) with positive culture of CR-GNB, or with febrile neutropenic and highly suspected infection of CR-GNB who used intravenous polymyxin B were first included in this study and then excluded according to the following exclusion criteria: 1) drug course of <3 days; 2) Patients who discontinue polymyxin B treatment and without adequate assessment of efficacy and nephrotoxicity for various reasons; and 3) children who only received the nebulizer and intrathecal administration (due to the low systemic absorption and the effect on nephrotoxicity cannot be determined and its efficacy cannot be compared with intravenous administration).

The relevant information of the included children was registered through the hospital electronic case system, including the patent epidemiologic data, including age, sex, underlying disease, surgical operation (endotracheal intubation, secretion drainage, debridement), basic information of polymyxin B (dose, frequency, duration of treatment, combination of other antibacterial drugs, combination of other potentially nephrotoxic drugs), infection-related indicators [microbiological culture results before and after drug administration, white blood cell (WBC), patient axillary temperature, C-reactive protein (CRP), procalcitonin (PCT)], and survival status at the time of discharge.

### Definition of Related Indicators

Microbiological results were assessed by repeated microbiological cultures at the end of treatment (two consecutive measurements within 1 week after the end of treatment). The VITEK^®^ 2 Compact automated microbial identification system (bioMérieux, France) was used for the identification of bacilli and for drug susceptibility testing, blood culture specimens are first grown in blood culture bottles to prepare bacterial particles and all other specimens were inoculated in agar plates for the identification and drug susceptibility tests. All pathogenic test results referred to the Standards 2017 of Clinical and Laboratory Standards Institute (CLSI) (CLSI et al., 2014)^.^Carbapenem resistance was defined as the minimum inhibitory concentration (MIC) value for imipenem and meropenem ≥8 mg/L. Based on the results of microbial culture, it was considered bacterial clearance if pathogenic bacteria were not present; it was considered microbial treatment failure if the original pathogenic bacteria were still present; it was considered secondary infection if new pathogenic bacteria were present; and it was considered presumptive bacterial clearance if the culture specimens could not be collected due to effective treatment (in some diseases, the disappearance of signs and symptoms makes culturable material inaccessible (e.g., sputum, skin pus, or secretions), or the method of obtaining specimens is too invasive for recovered patients).

Favorable clinical response was defined as successful microbial treatment, and the patient’s signs of infection, temperature, CRP, PCT, and WBC returned to normal at the end of treatment with polymyxin B (the last measurement results within 1 week after the end of treatment were used) or only one of them did not return to normal but decreased compared with pretreatment; Unfavorable clinical response was defined as failure of microbial treatment or secondary infection, and/or among the patient’s signs of infection, temperature, CRP, PCT, and WBC, one or two of them did not return to normal or deteriorated further (Beam et al., 1992; Smith et al., 1998; Li et al., 2021). An axillary temperature less than or equal to 37.5°C was considered normal. The serum PCT level was detected by electrochemiluminescence immunometric assay, and a level not exceeding 0.5 ng/ml was considered normal (Ding et al., 2020). The plasma CRP level was measured by immunoturbidimetry assay, and the reference value provided by the manufacturer was <10 mg/L. The WBC level was measured by flow cytometry according to the instructions of the manufacturer, and a WBC value of 3.5 × 10^9^/L-9.5 × 10^9^/L was considered normal.

The definition and staging of AKI were based on the RIFLE standards (Venkataraman and Kellum, 2007). A 1.5-fold increase in serum creatinine (SCr) or a >25% decrease in glomerular filtration rate (GFR) was considered at higher risk of developing kidney disease; a 2-fold increase in SCr or a 50% decrease in GFR was considered kidney injury; and a 3.3-fold increase in SCr or a 75% decrease in GFR was considered kidney failure. The incidence of AKI was calculated as the sum of the three and was determined by the maximum creatinine value measured during the course of drug administration.

Taking the effectiveness of the treatment and the incidence of AKI during drug administration as the main outcome indicators, the cases lacking data on the main outcome indicators were excluded, and all cases with complete data were included in the final analysis.

### Statistical Method

The data were statistically analyzed using IBM SPSS 24.0 software (IBM, United States). Normally distributed measures were expressed as the mean ± SD, and means were compared using the t-test. Nonnormally distributed counts were expressed as the median [interquartile range (IQR)], and nonnormally distributed data were tested using the Mann–Whitney U test. Count data were expressed as numbers and percentages and examined using Pearson’s chi-square test (χ2) or Fisher’s exact test. Factors influencing the effectiveness of polymyxin B and the incidence of AKI were analyzed by logistic regression in subgroup analysis, and variables found to be significantly associated with the clinical effectiveness or the incidence of AKI in the univariate analysis (*p* < 0.05) were entered into the multivariate logistic regression model for multivariate regression analysis. A *p* value below 0.05 (*p* < 0.05) was considered statistically significant.

## Results

A total of 63 children using intravenous polymyxin B in the hospital during the 2 years from June 2019 to June 2021 were found, and 8 cases were excluded after screening (4 cases with a course of treatment <3 days, 1 case with nebulizer administration alone, and 3 cases without adequate assessment of efficacy and nephrotoxicity for discontinue polymyxin B treatment for various reasons. Therefore, a total of 55 children were ultimately included in this study. Most (67.3%, 37/55) were male, and 16.4% (9/55) were under 1 year of age, with a mean age of 10 years. The demographic and clinical information of these children is summarized in Table 1. Bacterial culture and drug sensitivity tests confirmed that 53 patients were infected by CR-GNB, and a total of 71CR-GNB samples were isolated (37 patients had a single CR-GNB infection, 16 patients had 2 CR-GNB infections). and other 2 patients with febrile neutropenic and highly suspected infection of CR-GNB (Various antibiotics such as carbapenem, beta-lactam and tigecycline have been used before polymyxin B and all of which have poor efficacy) treated with polymyxin B. the majority of the organisms were isolated from the respiratory tract (92.7%, 51/55), followed by bloodstream infections (47.3%, 26/55) for CR-GNB infection, with *Klebsiella pneumoniae* (58.2%) being the most frequently isolated organism, followed by *Acinetobacter baumannii* (34.5%), *Pseudomonas aeruginosa* (20.0%) and *Escherichia coli* (9.1%), as shown in Table 1. Other isolated carbapenem resistance pathogens included *Klebsiella oxytoca* (*n* = 1), *Citrobacter rodentium* (*n* = 1), *Enterobacter cloacae* (*n* = 1) and *Chryseobacterium meningosepticum* (*n* = 1) (Table 1). Other isolated polymyxin B non-susceptible pathogens include *Staphylococcus* (*n* = 4), Stenotrophomonas *maltophilia* (*n* = 2), *Enterococcus faecalis* (*n* = 2), and Ralstonia mannitolilytica (*n* = 1), sensitive antibiotics were given at the same time during the treatment. All-cause mortality during hospitalization was 7.3% (4/55) (Table 1).

**TABLE 1:** Patient characteristics with favorable and unfavorable clinical responses during polymyxin B therapy.

Related indicators	Overall (N = 55)	Favorable clinical	Unfavorable clinical	*p* value
		Response (N = 29)	Response (N = 26)	
Demographics				
Age (Months) (mean ± SD)	120.55 ± 73.19	108.28 ± 74.19	134.23 ± 70.97	0.192
Age (≤1 year) (*n*, %)	9 (16.4)	6 (20.7)	3 (11.5)	0.360
Weight (kg) (Median, IQR)	38 (17, 50)	30 (14.25, 50)	41 (18, 50)	0.270
Gender (male) (*n*, %)	37 (67.3)	20 (69.0)	17 (65.4)	0.778
ICU admission (*n*, %)	34 (61.8)	19 (65.5)	15 (57.7)	0.551
Hospitalization days (Median, IQR)	37 (23, 51)	38 (25.5, 53.5)	33 (21.75, 51.5)	0.479
Comorbidities (*n*, %)				
Hypoproteinemia	26 (47.3)	12 (41.4)	14 (53.8)	0.355
Surgery	22 (40.0)	13 (44.8)	9 (34.6)	0.440
Pulmonary diseases	17 (30.9)	10 (34.5)	7 (26.9)	0.545
Electrolyte disturbance	28 (50.9)	11 (37.9)	17 (65.4)	0.042
Hypokalemia	17 (30.9)	6 (20.7)	11 (42.3)	0.083
Hyponatremia	8 (14.5)	4 (13.8)	4 (15.4)	0.867
Hypocalcemia	19 (34.5)	8 (27.6)	11 (42.3)	0.252
Hypomagnesemia	7 (12.7)	2 (6.9)	5 (19.2)	0.171
Other electrolyte disturbance	5 (9.1)	0 (0)	5 (19.2)	0.019
Blood disease	26 (47.3)	13 (44.8)	13 (50.0)	0.701
Heart disease	18 (32.7)	11 (37.9)	7 (26.9)	0.385
Central system disease	19 (34.5)	11 (37.9)	8 (30.8)	0.577
Digestive system disease	16 (29.1)	8 (27.6)	8 (30.8)	0.777
Viral infectious diseases	3 (5.5)	3 (10.3)	0 (0)	0.777
Trauma	8 (14.5)	6 (20.7)	2 (7.7)	0.777
Sepsis shock	24 (43.6)	11 (37.9)	13 (50.0)	0.368
Site of infection (*n*, %)				
Pneumonia	51 (92.7)	26 (89.7)	25 (96.2)	0.354
Bloodstream infection	26 (47.3)	14 (48.3)	12 (46.2)	0.875
Intraabdominal infection	5 (9.1)	3 (10.3)	2 (7.7)	0.733
Abdominal infection	2 (3.6)	1 (3.4)	1 (3.8)	0.937
Soft tissue infection	2 (3.6)	2 (6.9)	0 (0)	0.173
Urinary tract infection	3 (5.5)	2 (6.9)	1 (3.8)	0.619
Number of infected sites (Median, IQR)	2 (1,2)	2 (1,2)	2 (1,2)	0.492
Type of microorganism (CR-GNB) (*n*, %)				
*Klebsiella pneumoniae*	32 (58.2)	18 (62.1)	14 (53.8)	0.537
*Acinetobacter baumannii*	19 (34.5)	9 (31.0)	10 (38.5)	0.563
*Pseudomonas aeruginosa*	11 (20.0)	5 (17.2)	6 (23.1)	0.589
*Escherichia coli*	5 (9.1)	3 (10.3)	2 (7.7)	0.733
Other CR-GNB	4 (7.3)	1 (3.4)	3 (12)	0.232
Outcome (*n*, %)				
Hospital mortality	4 (7.3)	2 (6.9)	2 (7.7)	0.910
Microorganism Clearance	39 (70.9)	29 (100.0)	10 (38.5)	<0.001
Secondary infection	7 (12.7)	3 (10.3)	4 (15.4)	0.576
AKI	15 (27.3)	5 (17.2)	10 (38.5)	0.078
Risk stage	5 (9.1)	2 (6.9)	3 (11.5)	0.659
Injury stage	6 (10.9)	3 (10.3)	3 (11.5)	0.887
Failure stage	4 (7.3)	0 (0)	4 (15.4)	0.044

### Analysis of the Clinical Effectiveness

Twenty-nine (52.7%) and thirty-nine (70.9%) patients had favorable clinical and microbiological responses, respectively (Table 1). We compared the differences in characteristics between groups with clinically favorable and unfavorable outcomes, including demographics, comorbidities, underlying disease, site of infection and pathogenic bacteria, as shown in Table 1, and the polymyxin B dosing and concomitant drugs in patients, as shown in Table 2. The results showed only a few differences in clinical characteristics between the two groups, and the incidence of electrolyte disturbances was higher in the unfavorable group than in the favorable group (*p* = 0.042). However, there was no difference between the two groups in the subgroup analysis, as shown in Table 1. The treatment duration of polymyxin B therapy was longer in the favorable group than in the unfavorable group (*p* = 0.027), as shown in Table 2.

**TABLE 2:** Polymyxin B dosing and concomitant drugs in patients with favorable and unfavorable clinical response.

Related indicators	Favorable clinical response (N = 29)	Unfavorable clinical response (N = 26)	*p* value
Polymyxin B treatment			
Duration days (Median, IQR)	10 (7.5, 16)	8 (4.5, 10.5)	0.027
First loading dose (*n*, %)	11 (37.9)	8 (30.8)	0.577
Daily dose (mg/kg/d) (Median, IQR)	2 (2,3)	2 (2, 2.5)	0.327
<1.5 mg/kg/day (*n*, %)	0 (0)	2 (7.7)	0.128
1.5–2.5 mg/kg/day (*n*, %)	23 (79.3)	19 (73.1)	0.587
>2.5–4.0 mg/kg/day (*n*, %)	6 (20.7)	5 (19.2)	0.893
Cumulative (Total) dose (mg) (Median, IQR)	290 (141.5, 437.5)	250 (134.5, 425)	0.729
Concomitant antibiotic therapy^a^ (*n*, %)	24 (82.8)	25 (96.2)	0.112
Carbapenem	10 (34.5)	7 (26.9)	0.545
Tigecycline	4 (13.8)	1 (3.8)	0.200
Tigecycline + Carbapenem	1 (3.4)	7 (26.9)	0.014
other β-lactams^b^	2 (6.9)	6 (23.1)	0.089
Others	7 (24.1)	4 (15.4)	0.418
Concomitant potential nephrotoxic drugs (*n*, %)	14 (48.3)	13 (50.0)	0.898
Furosemide	8 (27.6)	8 (30.8)	0.795
Immunosuppressant	5 (17.2)	3 (11.5)	0.549
Nonsteroidal anti-inflammatory drugs	3 (10.3)	4 (15.4)	0.576
Amphotericin B	1 (3.4)	2 (7.7)	0.489
Concomitant other drugs (*n*, %)			
Antifungal drugs except amphotericin B	13 (44.8)	9 (34.6)	0.440
Glucocorticoid	10 (34.5)	14 (53.8)	0.148

a Antibiotic mainly used to treat Gram-positive bacterial infections are not included, such as linezolid, vancomycin, teicoplanin etc.

b Include cefoperazone/sulbactam, Piperacillin/tazobactam and cefepime.

Furthermore, we performed logistic regression analysis on these two indicators, while multivariate logistic regression analysis showed that neither of these factors was a predictor of treatment success with electrolyte disturbance (OR 0.34; 95% CI 0.11–1.053; *p* = 0.061) or duration of treatment with polymyxin B (OR 1.078; 95% CI 0.981–1.186; *p* = 0.120) (Table 3).

**TABLE 3:** Univariable and multivariable logistic regression analysis of a favorable clinical outcome.

Variable	Unadjusted OR (95% CI)	*p* value	Adjusted OR (95% CI)	*p* value
Electrolyte disturbance	0.324 (0.107, 0.974)	0.045	0.34 (0.11, 1.053)	0.061
Duration days of Polymyxin B treatment	1.085 (0.987, 1.192)	0.09	1.078 (0.981, 1.186)	0.12

### Analysis of the Incidence of Acute Kidney Injury During Treatment

AKI was observed in 27.3% (15/55) of patients with risk stage 9.1% (5/55), injury stage 10.9% (6/55) and failure stage 7.3% (4/55). No patients adjusted the dose of polymyxin B due to AKI. Most patients (13/15) recovered or improved renal function 1 week after polymyxin B was discontinued, and only two patients who progressed to failure stage had further deterioration of renal function. The characteristics of patients with AKI (*n* = 15) and non-AKI (*n* = 40) during treatment with polymyxin B are shown in Supplementary Tables S1, S2. Furthermore, we revealed concomitant glucocorticoids (OR = 9.102, 95% CI 1.899, 43.631, *p* = 0.006) to be associated with the onset of AKI after adjusting for underlying confounders through multivariate analysis (Supplementary Table S3).

## Discussion

In this study, a retrospective analysis of the clinical data of 55 children treated with intravenous polymyxin B in a large teaching hospital in China was conducted, and the results showed that the effective rate of using polymyxin B in the treatment of CR-GNB infection in children was 52.7%. Most of the antibacterial treatment regimens based on polymyxin B were combined with other antibacterial drugs (82.8%). The duration of treatment with polymyxin B may affect its anti-infective efficacy, and the course of treatment was longer in the favorable clinical outcome group than in the unfavorable outcome group (10 vs. 8, *p* = 0.027). Electrolyte disturbances in children during their use may lead to unfavorable clinical outcomes (*p* = 0.042). However, potential factors affecting clinical outcomes were not identified in the multifactorial analysis, probably due to the small sample size. The risk of AKI during treatment with polymyxin B was 27.3%, but severe AKI was only 7.3%, and most children tolerated polymyxin B and continued treatment, with an all-cause mortality rate of 7.3% at hospital discharge.

The results of this study showed that the efficiency of using polymyxin B for the treatment of severe infections in children was 52.7%, which was lower than that of Siddiqui et al. (57.1%) (Siddiqui et al., 2014). This could be related to the fact that the children in Siddiqui’s study were treated with a higher dose of polymyxin B (all were given a dose of 40, 000 IU/kg/d, equal to 4 mg/kg/d), whereas the majority of cases in this study were given the FDA’s recommended daily dose of 1.5–2.5 mg/kg/day (76.4%) (Chlossberg and Samuel, 2017). Another retrospective study conducted by Saleem et al. (2011) included eight children who were also given a high dose of 4 mg/kg/d polymyxin B, and all patients achieved microbiological clearance at the end of treatment (no clinical efficacy was reported). Therefore, we speculate that an appropriate increase in the dose of polymyxin B may improve its clinical efficacy and the risk of AKI at the same time. PK/PD studies on polymyxin B in adults have shown that the free area under the concentration-time curve (fAUC)/minimal inhibitory concentration (MIC) is the PK/PD index that best predicts polymyxin B activity (Cheah et al., 2015; Landersdorfer et al., 2018). Therefore, an appropriate increase in the dose of polymyxin B is beneficial for achieving higher PK/PD target values and enhancing clinical effectiveness. Some preliminary studies in adults have also shown that an appropriate increase in the dose of polymyxin B is beneficial for clinical effectiveness (Elias et al., 2010).

A review and meta-analysis conducted by Falagas and his coworkers showed that the all-cause nephrotoxicity of 2994 adult patients (from 28 studies) treated with intravenous polymyxin B was 40.7% (Falagas et al., 2021), and our study showed a lower incidence of AKI in children with polymyxin B than in adult patients at 27.3%, closer to the data reported by Siddiqui et al. on children (21.4% incidence of nephrotoxicity) (Siddiqui et al., 2014). However, a meta-analysis of the clinical data of 405 children treated with colistin showed that the incidence of nephrotoxicity in children treated with colistin was 6.1% (Karageorgos et al., 2019), much lower than that in our study. This may be related to the different criteria used to determine nephrotoxicity in our study, as two of the studies on colistin (Falagas et al., 2009; Kapoor et al., 2013) used an increase in creatinine greater than two times the baseline as a criterion for AKI, and our study used the maximum creatinine level during dosing to determine the occurrence of AKI (may amplify the potential nephrotoxicity of polymyxin B). Current studies (Thomas et al., 2019) have shown that colistin is mainly given in the form of its inactive prodrug, colistin methane sulfonate (CMS), which requires hydrolysis to activate colistin *in vivo*. This conversion is incomplete, slow, and unpredictable compared to polymyxin B, which is given directly in its active form and does not undergo conversion *in vivo*, allowing for faster and higher steady-state concentrations. A comparative analysis based on the US Food and Drug Administration Adverse Event Reporting System (FAERS) database showed a lower incidence of AKI in adults treated with polymyxin B than with colistin (Truong et al., 2021), and a meta-analysis using RIFLE as a criterion for kidney injury also showed a 37% increased risk of nephrotoxicity in the colistin treatment group compared to the treatment with polymyxin B group (RR = 1.37, 95% CI: 1.13–1.67) (Sisay et al., 2021). The results of this study are inconsistent with the data on adults, it may be related to the small sample size of this study, and further large-sample studies are needed to explore.

No patients in this study had dose adjustments of polymyxin B due to AKI. Although FDA recommended that the dose should be reduced from 1.5 mg/kg downward for individuals with kidney impairment and the total daily dose must not exceed 2.5 mg/kg (Chlossberg and Samuel, 2017). Guidelines in recent years have recommended that polymyxin B does not require dose adjustment due to AKI based on PK/PD considerations for polymyxin B is mainly metabolized by non-renal pathways (Tsuji et al., 2019; Yang et al., 2021). However, since children cannot express subjective symptoms of poisoning, it is recommended that close clinical monitoring should be performed.

A study conducted by Wacharachaisurapol and his coworkers showed no difference in the incidence of AKI in children receiving a loading dose of intravenous colistin compared with that in children receiving no loading dose, so a loading dose should be prescribed to achieve better drug exposure necessary for the treatment of multidrug-resistant Gram-negative bacteria (Wacharachaisurapol et al., 2021). Our results also showed that administration of a loading dose of polymyxin B did not increase the risk of AKI. However, the loading dose also did not increase the efficacy of the treatment. Although our findings show the proportion of patients given a loading dose was greater than in the nonloading dose group in the AKI (40 vs. 32.5%, *p* = 0.602) and the effective treatment groups (37.9 vs. 30.8%, *p* = 0.577), respectively. And their differences were not statistically significant may due to the small sample size. Therefore, the effect of loading dose on the AKI and the efficacy for CR-GNB infection in children needs to be confirmed by large-scale clinical studies in future.

The results of this study showed that the combination of glucocorticoids is an independent risk factor for AKI in children treated with polymyxin B, so that the children treated with polymyxin B in combination with glucocorticoids need closer monitoring of kidney function. At present, among the risk factors for AKI caused by the use of polymyxin B in adults and by the use of colistin in children, the combination of glucocorticoids has not been found to affect polymyxin B-induced AKI (Zeng et al., 2020; Zhang et al., 2021), and future attention is needed. In addition, our results showed that the frequency of electrolyte disturbances (65.4 vs. 37.9%, *p* = 0.042) and severe renal injuries (15.0% vs. 0, *p* = 0.044) were higher in the ineffective treatment group than in the effective treatment group, suggesting that the incidence of electrolyte disturbances and severe renal injuries may affect the clinical outcome of children. Studies on the use of colistin in children also showed that newborns might experience electrolyte disturbances due to the use of colistin (Serafettin Tekgunduz et al., 2015; Al-Lawama et al., 2016), and the results of this study indicated that similar problems might occur with polymyxin B. Therefore, the monitoring of electrolyte levels and renal function should be strengthened during the use of polymyxin in children.

The limitations of this study are as follows: 1) it was a single-center retrospective study, and there may be some sample selection bias; and 2) the sample size of this study was small, so the statistical efficacy was low.

## Conclusion

Polymyxin B can be used as a salvage therapy for severe CR-GNB infection in children when no other susceptible antibiotics are available, and should be alert to its higher incidence of AKI and strengthen the monitoring of kidney function at the same time.

## Data Availability

The original contributions presented in the study are included in the article/Supplementary Material, further inquiries can be directed to the corresponding authors.
