# Supplementary material for: Effectiveness and Nephrotoxicity of Intravenous Polymyxin B in Carbapenem-Resistant Gram-Negative Bacterial Infections Among Chinese Children
Source: Front Pharmacol. 2022 May 27;13:902054. doi: 10.3389/fphar.2022.902054 (PMC9197179; doi:10.3389/fphar.2022.902054)
Supplement: Supplementary file 1 [file DataSheet1.docx]

**Supplementary tables**

Table S1 Patient characteristics with and without AKI occurring during polymyxin B therapy

| **Related indicators** | **AKI occurs (N=15)** | **AKI did not occur (N=40)** | **P value** |
| --- | --- | --- | --- |
| **Demographics** |  |  |  |
| Age (Months) (mean ± SD) | 139.52 ± 66.917 | 113.43 ± 74.965 | 0.242 |
| Age (≤1 years) (n, %) | 1 (6.7) | 8 (20.0） | 0.234 |
| Weight (Median, IQR) | 40 [18, 50] | 33 [12, 50] | 0.402 |
| Gender (male) (n, %) | 11 (73.3) | 26 (65.0) | 0.557 |
| ICU admission (n, %) | 9 (60.0) | 25 (62.5) | 0.865 |
| Hospitalization days (Median, IQR) | 29 [20, 56] | 37 [26, 51] | 0.321 |
| **Comorbidities (n, %)** |  |  |  |
| Hypoproteinemia | 9 (60.0) | 17 (42.5) | 0.247 |
| Surgery | 6 (40.0) | 16 (40.0) | 1.000 |
| Pulmonary diseases | 4 (26.7) | 13 (32.5) | 0.677 |
| Electrolyte disturbance | 9 (60.0) | 19 (47.5) | 0.409 |
| Blood disease | 8 (53.3) | 18 (45.0) | 0.581 |
| Heart disease | 7 (46.7) | 11 (27.5) | 0.177 |
| Central system disease | 4 (26.7) | 15 (37.5) | 0.452 |
| Digestive system disease | 6 (40.0) | 10 (25.0) | 0.275 |
| Infectious diseases | 1 (6.7) | 2 (5.0) | 0.808 |
| Trauma | 1 (6.7) | 7 (17.5) | 0.310 |
| Sepsis | 11 (73.3) | 13 (32.5) | 0.007 |
| **Outcome (n, %)** |  |  |  |
| Hospital mortality | 0 (0) | 4 (10.0) | 0.203 |

Table S2 Polymyxin B dosing and concomitant drugs in patients with and without AKI

| **Related indicators** | **AKI occurs (N=15)** | **AKI did not occur (N=40)** | **P value** |
| --- | --- | --- | --- |
| **Polymyxin B treatment** |  |  |  |
| Duration days (Median, IQR) | 8 [4, 15] | 10 [7, 15] | 0.178 |
| First loading dose (n, %) | 6 (40.0) | 13 (32.5) | 0.602 |
| Daily dose (mgkg/d) (n, %) | 2 [2, 2] | 2 [2, 3] | 0.485 |
| ＜1.5 mg/kg/day (n, %) | 1 (6.7) | 1 (2.1) | 0.475 |
| 1.5-2.5 mg/kg/day (n, %) | 12 (80.0） | 30 (75.0) | 1.000 |
| ＞2.5-4.0 mg/kg/day (n, %) | 2 (13.3) | 9 (22.5) | 0.708 |
| Cumulative (Total) dose (mg) (Median, IQR) | 270 [130, 450] | 254 [150, 425] | 0.902 |
| **Concomitant drugs (n, %)** |  |  |  |
| Combine with other antibacterial drugs | 14 (92.3) | 37 (92.5) | 0.916 |
| Tigecycline | 6 (40.0) | 11 (27.5) | 0.372 |
| β-lactam | 4 (26.7) | 10 (25.0) | 0.899 |
| Carbapenem | 8 (53.3) | 22 (55.0) | 0.912 |
| Fosfomycin | 1 (6.7) | 4 (10.0) | 0.702 |
| Aminoglycoside | 2 (13.3) | 3 (7.5) | 0.503 |
| Vancomycin | 1 (6.7) | 5 (12.5) | 0.537 |
| Sulfonamide | 2 (13.3) | 1 (2.5) | 0.115 |
| Teicoplanin | 3 (20.0) | 2 (5.0) | 0.085 |
| Linezolid | 5 (33.3) | 3 (7.5) | 0.016 |
| Types of combined antibacterial drugs | 1 [1, 2] | 2 [1, 3] | 0.055 |
| **Potential nephrotoxic drugs (n, %)** | 8 (53.3) | 19 (47.5) | 0.700 |
| Furosemide | 6 (40.0) | 10 (25.0) | 0.275 |
| Immunosuppressant | 4 (26.7) | 4 (10.0) | 0.118 |
| Nonsteroidal anti-inflammatory drugs | 3 (20.0) | 4 (10.0) | 0.322 |
| Amphotericin B | 1 (6.7) | 2 (4.2) | 0.808 |
| **Concomitant other drugs (n, %)** |  |  |  |
| Antifungal drugs except amphotericin B | 7 (46.7) | 15 (37.5) | 0.537 |
| Glucocorticoid | 12 (80.0) | 12 (30.0) | 0.001 |

Table S3 Univariable and multivariable logistic regression analysis of acute kidney injury (AKI)

| **Variable** | **Unadjusted OR (95% CI)** | **P value** | **Adjusted OR (95% CI)** | **P value** |
| --- | --- | --- | --- | --- |
| Sepsis shock | 5.712 (1.523, 21.421) | 0.010 | 4.144 (0.896, 19.164) | 0.069 |
| Linezolid | 6.167 (1.254, 30.322) | 0.025 | 3.435 (0.528, 22.329) | 0.196 |
| Glucocorticoid | 9.333 (2.223, 39.179) | 0.002 | 9.102 (1.899, 43.631) | 0.006 |
